# Attending to power differentials: How NP‐led group medical visits can influence the management of chronic conditions

**DOI:** 10.1111/hex.12525

**Published:** 2017-01-10

**Authors:** Laura Housden, Annette J. Browne, Sabrina T. Wong, Martin Dawes

**Affiliations:** ^1^ School of Nursing University of British Columbia Vancouver BC Canada; ^2^ Centre for Health Services and Policy Research University of British Columbia Vancouver BC Canada; ^3^ CRiHHI: Critical Research in Health and Healthcare Inequities University of British Columbia Vancouver BC Canada; ^4^ Department of Family Practice Faculty of Medicine Vancouver BC Canada

**Keywords:** chronic disease, diffusion of innovation, group medical visits, nurse practitioner, power, quality of care

## Abstract

**Objective:**

In Canada, primary care reform has encouraged innovations, including nurse practitioners (NPs) and group medical visits (GMVs). NP‐led GMVs provide an opportunity to examine barriers and enablers to implementing this innovation in primary care.

**Design:**

An instrumental case study design (n=3): two cases where NPs were using GMVs and one case where NPs were not using GMVs, was completed. In‐depth interviews with patients and providers (N=24) and 10 hours of direct observation were completed. Interpretive descriptive methods were used to analyse data.

**Results/Findings:**

Two main themes were identified: (i) acquisition of knowledge and (ii) GMVs help shift relationships between patients and health‐care providers. Participants discussed how patients and providers learn from one another to facilitate self‐management of chronic conditions. They also discussed how the GMV shifts inherent power differentials between providers and between patients and providers.

**Discussion:**

NP‐led GMVs are a method of care delivery that harness NPs’ professional agency through increased leadership and interprofessional collaboration. GMVs also facilitate an environment that is patient‐centred and interprofessional, providing patients with increased confidence to manage their chronic conditions. The GMV provides the opportunity to meet both team‐based and patient‐centred health‐care objectives and may disrupt inherent power differentials that exist in primary care.

## 1 INTRODUCTION

Primary care reform is aimed at strengthening the health‐care system;^1^ growing evidence suggests that stronger primary care contributes to healthier populations.^2^, ^3^ Innovative approaches to primary care reform in Canada can include changing the ways in which patients interact with their providers and each other such, as in the case of group medical visits (GMVs) or shared medical appointments between patients.^4^ Other innovations are aimed at broadening the health‐care team to include various providers such as nurse practitioners (NPs).^5^

GMVs enable health‐care providers to work together to deliver services to patients in a group format, rather than the conventional single‐patient, single‐provider format.^6^ While different types of GMVs exist (eg drop‐in groups based on a common issue such as pain or women's wellness),^6^, ^7^, ^8^, ^9^ the most common type of GMV is appointments for patients with a shared medical condition. During the GMV, patients meet for their primary care appointment(s) together and receive services in a group environment.^6^ The GMV includes a review of recent laboratory results, an education piece that often focuses on aspects of a shared chronic condition, an interactive discussion^10^, ^11^, ^12^, ^13^, ^14^, ^15^, ^16^, ^17^ and the delivery of preventative or health promotion health services.^6^, ^18^ Past work has shown that confidentiality is not a major concern for patients attending GMVs; rather, they increase patient and provider trust.^19^ GMVs have been used with success in Canada,^4^, ^20^ the USA,^4^, ^21^, ^22^, ^23^, ^24^ Europe^10^, ^11^, ^12^, ^13^, ^15^, ^16^ and China.^17^

In North America, NPs are advanced practice nurses (APNs) who have completed postgraduate level training and function in an expanded scope of nursing practice.^25^, ^26^, ^27^ The scope of NP practice includes the ability to diagnose diseases, prescribe medications, order and interpret laboratory tests and refer patients to specialists.^25^ In Canada, NPs often work as part of interprofessional teams.^28^

Unfortunately, the combined use of NP‐led GMVs in primary care remains limited in Canada. This has been hampered by a number of factors, some of which are structural such as the dominant remuneration model of fee‐for‐service^29^, ^30^ and the availability of an appropriately large clinical space. Some of the barriers to the implementation of NP‐led GMVs are interpersonal such as individual provider capabilities.^31^ Whether innovations are implemented or not can be influenced by power differentials.^32^ In the case of NP‐led GMVs, power differentials exist between patients and providers and between different health professions (eg medicine, nursing, pharmacy). While there is substantial research on the area of NP practice, there is scant research on NP‐led GMVs^33^, ^34^ and no published research on NP‐led GMVs in Canada.

The existence of power differentials contributes to complexity in any given work environment, health care notwithstanding. Power differentials between health‐care professionals are accentuated by organizational constraints (eg policies and procedures, health‐care budgets, staff privileges)^35^ (p. 117) and the fact that individuals have varying levels of “agency” or individual power and authority.^36^ Physicians typically have more power and authority than nurses given their scope of practice and because most are considered independent contractors who bill the public insurer (provincial government) for their services.^37^ In most conventional practice settings, physicians provide the diagnosis, medical treatment and a course of treatment for care.^37^, ^38^, ^39^ Nurses, while responsible for the care they deliver, are typically employees of organizations, which in some situations include private physician practices. With the adoption of NPs, the role and scope of nurses’ practice in primary care have shifted such that NPs have an overlapping scope of practice with their family physician colleagues.^5^, ^40^, ^41^, ^42^ While the scope of practice for nurses and NPs has changed, the ways in which health professions work together has been slower to change.^43^ Little is known as to how power differentials influence the diffusion of innovations in primary care. The purpose of this study was to examine NP‐led GMVs for patients with chronic conditions and consider the barriers and enablers to implementing GMVs in one Canadian province, British Columbia.

## 2 METHODS

### 2.1 Study design

The results reported are part of a larger study that used a case study design consisting of three cases based in both urban and rural British Columbia (BC). The instrumental case study design was used to provide broader understanding of the phenomenon (NP‐led GMVs).^44^ Case study approaches are appropriate when studying “complex subjects within their context”^45^ and allow a rich, in‐depth understanding of the study phenomenon to develop.^46^ Recruitment of participants occurred over 12 months between January 2013 and January 2014. After 1 year of recruitment, a total of 24 patients and health‐care providers participated in in‐depth interviews and 10 hours of direct observation was completed for a total of three case studies.

Cases 1 and 2 included primary care practices where NPs led GMVs. The first case included a primary care practice where the NP organized and administered a GMV focused on healthy nutrition for patients with various chronic conditions, including obesity, diabetes and heart disease. The second case involved a primary care practice where the NP with support from an interdisciplinary health‐care team offered GMVs about diabetes management. In case 2, other health‐care providers also attended the answer patients’ questions and assist with prescriptions.

The primary researcher (LH) observed seven GMVs, totalling 10 hours of direct observation. Two GMVs were observed in case 1, and five GMVs were observed in case 2. The number of patients attending each GMV varied, from 12 to 28 patients in attendance. For cases 1 and 2, both patients who attended the GMVs (n=12) and health‐care providers (n=5) were interviewed. We also sought to interview patients who had been invited to attend GMVs but declined to participate. However, information on who had been invited was not available, and patients did not respond to the recruitment poster placed at the clinics. The boundaries of cases 1 and 2^47^ were the primary care clinics.

In cases 1 and 2, patients received health‐care assessments at the beginning of the GMV. This included blood pressure, weight and foot checks as necessary. When all participants arrived, the NP reviewed procedures keeping information confidential and introduced the topics for discussion. In both cases, the NP guided the discussion with participants, clarifying misconceptions and encouraging patients to share goals and health challenges in the day‐to‐day management of their chronic condition(s). In case 2, one of the other health‐care providers would also provide additional information or clarity as needed. At the completion of the group, goals and discussion topics for the next session were determined. The health‐care provider(s) remained in the group to answer any remaining questions at the end of each session. In case 1, patients could choose to individually discuss their laboratory results, but overall laboratory result as well as weight trends of the group was discussed. In case 2, patient's laboratory results were shared on a large white board at the front of the room.

Case 3 included NPs (n=7) who were not leading GMVs, but were willing to discuss their ideas about GMVs. Nurse practitioners in the third case self‐identified as being primary care providers, although their clinical practices and target populations varied including refugee health, mental health and addictions and student health.

We had initially sought to follow a NP who was implementing a GMV. After 6 months of recruitment, we were unable to find a NP available or supported to implement a GMV. This third case examined factors shaping decisions related to NPs not being able to offer GMVs in primary care. The boundary for the third case was defined by the geographic boundaries of two BC health authorities.

The research team created an initial list of NP‐led GMVs in British Columbia, Canada. Sampling for the cases was purposeful and theoretical.^48^, ^49^ For cases 1 and two, we purposely sought practices where NPs were offering GMVs. Our early analysis indicated NPs had challenges diffusing innovations such as GMVs in primary care, thus we identified the need for adding a third case of NPs who were not implementing GMVs. A clear audit trail was maintained throughout the study, including a case study protocol and database of case study documents. A conceptual diagram of the case study design is available as Figure S1.

### 2.2 Eligibility criteria and procedures for case 1 and case 2

Inclusion criteria for interviewing patients in cases 1 and 2 were as follows: adults aged 18‐80 years old who were English speaking and had attended GMVs for one or more chronic condition. Patients were required to have attended a NP‐led GMV at least twice in the past 12 months. Inclusion criteria for providers were involvement in at least two GMVs in the past 12 months. Health‐care providers offering GMVs were asked whether a research team member could attend and observe the medical appointment.

All patients who were attending upcoming GMVs were mailed information about the study by the clinic prior to one of the research team attending their GMV. Consent to observe the GMV was obtained from all patients immediately prior to direct observation. During direct observation, data were gathered via detailed field notes to better understand how the GMV functioned including data on the physical space, format of the GMV, interpersonal interactions amongst patients and between patients and health‐care providers, body language, roles, participation and presentation and discussion styles.

After attendance at the GMV, the primary researcher remained at the clinic to discuss the project and gather contact information for patients and health‐care providers interested in participating in in‐depth interviews to share their experiences. Interested participants were screened for eligibility and given the opportunity to participate via phone or in person, depending on geographic location. Interested participants were contacted a maximum of three times to complete an interview. Interview questions were open‐ended and designed to examine patient and health‐care provider perspectives on how GMVs with NPs could impact both patient's health and the broader clinical environment.

### 2.3 Eligibility criteria and procedures for case 3

Inclusion criteria for the third case were NPs practicing in primary care, living in one of two BC Health Authorities (one urban and one rural) and not currently facilitating GMVs. Email inquiries were sent to NPs in two health authorities through publically available contact information. The study was introduced, and NPs were asked whether or not they were facilitating GMVs to deliver care to patients. Nurse practitioners were contacted a maximum of three times to participate in the study if they were not using GMVs. The Ethics Boards of the University of British Columbia and the two health authorities where the NPs worked approved all procedures. A description of the cases and patient participants can be found in Table 1.

**Table 1:** Description of cases

Case no. 1: NP‐led GMV		Case no. 2: Interdisciplinary GMV		Case no. 3: No GMV case
Description of case				
A primary care practice in a rural BC community where the NP provides healthy living and nutrition‐focused GMVs. Clients attend GMV with a variety of chronic conditions including diabetes, obesity, heart disease and arthritis.		A primary care practice in a large urban centre where the NP works with a team of health‐care providers to offer GMVs, including a pharmacist, physician and patient volunteers. Clients attend GMVs for diabetes.		A case consisting of NPs from BC, working in primary care with patients who have chronic conditions and who are not offering GMVs in their practice.
Direct observations: two GMVs, 3 h total.		Direct observations: five GMVs, 7 h total.		
Total ParticipantsN=61=Health‐care provider		Total ParticipantsN=114=Health‐care providers		Total ParticipantsN=7
Patient demographics				
	N		N	
Patients	5	Patients	7	
Female	4	Female patients	3	
Age (y)				
40‐44	1	65‐69	2	
55‐59	2	70‐74	1	
60‐64	2	75‐79	1	
		Declined	3	
Patient ethnicity				
Euro‐Canadian	4	Euro‐Canadian	6	
Metis	1	Metis	1	
Family context				
Married lives with partner	4	Married lives with partner	1	
Never married	1	Divorced	2	
		Separated	1	
		Widowed	1	
		Declined	2	
Highest education				
Grade 12/GED	3	Grade 12/GED	4	
Diploma/Degree	2	Diploma/Degree	3	
Income				
$30 000‐$39 000	1	$20 000‐$29 000	3	
$40 000‐$49 000	1	$70 000‐$79 000	1	
$70 000‐$79 000	3	$90 000‐$99 000	1	
		Declined	2	
Employment				
Working part‐time	1	Working part time	2	
Working full‐time	1	Retired	4	
Retired	2	Unemployed	1	
Receiving disability payments	1			

### 2.4 Data analysis

All interviews were audio‐recorded, and both interviews and field notes from the direct observation were transcribed. These transcript data were organized using NVivo.^50^ Interpretive descriptive methods were used to analyse the data.^51^ Data were first organized into broad conceptual categories, using deductive and inductive approaches. These broad categories were discussed in‐depth and validated amongst the research team. Similarities and differences in the data, both between and within cases, were considered. Data were aggregated into themes, and patterns and relationships between the data and each case were examined through the use of concept mapping.^52^ Each case was mapped by outlining the themes and considering patterns and relationships between and within the cases. Concept maps were discussed amongst the research team members. Nurse practitioner respondents were consulted after the data analysis to discuss and verify the findings.

### 2.5 Theoretical perspectives informing the data analysis

This study used both diffusion of innovation^53^ and theoretical perspectives on power^54^ as lenses from which to analyse the data. Diffusion of innovation theory has been applied in many health‐care situations.^55^, ^56^, ^57^, ^58^ Diffusion of innovation theory generally seeks to examine how a particular innovation is diffused over time within a social system.^53^ In this study, diffusion of innovation theory was used to examine NP‐led GMVs in primary care. Our initial analysis led to the recognition of power as a central concept influencing GMVs. To further examine these results, our analysis was informed by Foucaultian understandings of power and how it operates to make individuals “subject to someone else by control and dependence, and to his [sic] own identity by a conscience or self‐knowledge”^54^ (p. 212). Foucault's suggestion that the acquisition of knowledge serves to “intensify the exercise of power”^59^ (p. 35) supported an examination of the power differentials in health care and GMVs in particular. These power differentials have traditionally situated nurses as having “less authority” and knowledge than physicians^37^ and have potentially served as an oppressive force to the diffusion of health‐care innovations.

## 3 RESULTS

The analysis of the data resulted in the identification of two main themes relating to the following: (i) acquisition of knowledge and (ii) GMVs help shift relationships between patients and health‐care providers. Participants discussed how patients and providers learn from one another to facilitate the self‐management of chronic conditions. They also discussed how the GMV shifts inherent power differentials between providers and between patients and providers.

### 3.1 Acquisition of knowledge

Both patients and health‐care providers described how GMVs allowed for the acquisition of knowledge. This knowledge was acquired through increased understanding of how experiences are shaped by environment, geography, community and other social determinants of health. Foucault describes a process by which individuals become subjects through a process of control and dependency as well as a process of who the individuals’ “understand themselves to be”^60^ (p. 90). This process is deeply connected to the concept of knowledge and power.^59^ Through the acquisition of health and interpersonal knowledge, patients attending GMVs in cases 1 and 2 were able to harness more agency, that is, personal power and authority. For example, patients described how they gained more insight into the disease management process. This quote by a patient reflects how the GMV improved their knowledge and subsequently their ability to engage in self‐management. “… I'm actually managing. Even though its 10 years and things are supposed to get more difficult or get worse, I'm actually managing better. [I'm] more intelligent in managing things instead of acting out of fear” (Patient Interview #2).

#### 3.1.1 Increased knowledge about the context of individuals’ lives

A subtheme was that GMVs provided a space where providers and patients felt more connected to one another as there was increased sharing of knowledge about each other's lives. The building of relationships through GMVs contributed to a more in‐depth understanding of patients’ lives and health‐care providers’ daily work realities. This patient describes how the GMV moved beyond a medical appointment to become a space where individuals feel accepted and supported. “The group, it's a community. People aren't selected; they're just there and we, we just have to help each other as best as we can as a community and nobody wants to be alone with diabetes. They don't have to be alone” (Patient Interview #2). As this health‐care provider points out, GMVs were valuable in understanding the context of their patients. Moreover, GMVs encourage providers to learn how each provider interacts with each other. “…I feel like I know [patients] a little bit more. You might learn more about their life, or their family or their pastimes and hobbies…you know their social determinants of health; it's something that comes out a bit more….” (Healthcare Provider Interview #10).

This environment of shared understanding and a sense of community contributed to a shift in the traditional power dynamics. In many conventional primary care settings, health‐care providers are viewed as the “expert” and patients are supposed to “follow the [healthcare provider's] orders”.^61^, ^62^, ^63^ However, the acquisition of contextual knowledge gave GMV participant's additional agency and provided opportunities for patients to support each other and better self‐manage their chronic conditions.

#### 3.1.2 More knowledge equals more power

Another subtheme was that GMVs could broaden patient's perspectives of their chronic conditions. Not only were patients obtaining information from other patients on their health and chronic condition(s), but the group provided them with first‐hand accounts of how their disease could progress. Patients described these first‐hand accounts as helpful and motivating and new ways of learning developed through a process of observation and engagement with other patients. Patients recognized they were able to support each other to better self‐manage their chronic condition(s): “You get moral support from people who are also going through what you're going through, or even people who have it worse off than you, you know they have diabetes…you can look and go ‘Oh my God, I'm heading there..I gotta smarten up’” (Patient Interview #5). Both this quote and the one below from a health‐care provider indicate a realization of how each person has the opportunity to acquire more knowledge through the GMV. It also demonstrates how the group can encourage and motivate each other. The quote above also suggests that some patients might experience heightened anxiety with more knowledge about the disease progression. However, the GMV also provided a space for participants to see their contributions to the care of others and to hold each other accountable for improving their self‐care abilities.

I think because they are hearing it from more than just one, I think that when they see other people who are struggling with the same things that they are struggling with, it makes the situation come alive..and then when they see the great success that comes with everyone sharing the success, I think there's more of a buy‐in to make those changes.

(Healthcare Provider Interview #8)

The interpersonal interactions in the GMV also contributed to increased learning about the day‐to‐day management of chronic conditions, including a more in‐depth understanding of laboratory values and the potential complications of their condition(s):

I pay more attention to my chart now, more often since starting this group. Like my A1C was this number last month, now it's a different number this month and, like your kidney, your A1C, your HDL. I'm paying more attention to that more.

(Patient Interview #7)

Patients also noted how being with other patients in the group and observing the interaction between patients and health‐care providers often provided answers to questions they had regarding their health conditions. Observing this discussion allowed patients who may not have wanted to ask questions the opportunity to listen and receive answers.

…if you have a question about something you can bring it up they will discuss it. Someone will research it and bring the evidence. Generally if someone brings up a question other people will have the same question, only they haven't brought it up….You find out that some people, someone else brings something up and they say “oh yah, that's right.

(Patient Interview #8)

### 3.2 GMV helps shift relationships between patients and health‐care providers

Patients acknowledged that in most conventional health‐care settings, health‐care providers were in a position of power relative to the “average” person. “They [GMVs] are great. Especially for people in my age group or even older, sort of the relationship between medical professionals and myself, who is sort of an average Joe…they [Healthcare providers] are on a different level” (Patient Interview #8). Health‐care providers also discussed power differentials in primary care and noted how the GMV transformed the clinical encounter into a more patient‐centred approach. As this health‐care provider described:

…often the personality of a [healthcare provider] is they want to be in charge and they know best and they kind of want to be directing what happens, in most groups, that doesn't happen. The [healthcare provider] sits down and they're a member of the group and the discussion, but it's not the same level, [with] the patient and the other healthcare providers below, which was the old system. It really is the patient in the middle surrounded by all the healthcare professionals that are looking after the patients…

(Healthcare Provider Interview #9)

The environment of the GMV provided increased opportunities for patients and health‐care providers to engage with one another. GMV participants shared their personal challenges, successes and goals. This sharing fostered an environment in which patients felt as though their health‐care providers were also gaining valuable knowledge from the GMV. The two quotes below show that this change from the conventional health‐care provider/patient relationship served to humanize health‐care providers as individual's with their own challenges, burdens and health‐care goals. “You know, she's in our shoes, she's been in our shoes, she lives by the way she is teaching us” (Patient Interview #5).

They [healthcare providers] learn from us too, surprisingly. They learn quite a bit from us. The [doctor] wasn't eating lunch for a long time. We had a side bet, I'd stop some of my sugar intake and drink more water, she'd try to eat healthier lunches or veggies.. each time we'd check in with each other…

(Patient Interview #6)

Through this changed communication process, patients and health‐care providers described new ways of engaging in ways that acknowledged each person's particular contexts.

#### 3.2.1 Increasing personal agency

Group medical visits also enabled patients to be more in control of access to primary care. Patients acknowledged that their health‐care providers were busy and working in constrained environments (eg, 15‐minute visits to discuss only one problem). Yet, they described how attending the GMV relieved some of their perceived need to access traditionally delivered primary care services. This excerpt from a patient interview describes the discomfort at the number of times she previously accessed primary care and how the GMV provided confidence that she could go to the GMV as often as she wanted and that someone was monitoring her health on an on‐going basis:

My doctor is awesome, but I almost feel embarrassed about the number of times I kept going, and so, this sort of alleviates that a little bit, you see that it is okay, somebody is watching out for me in a general way as well.

(Patient Interview #1)

This patient's experience below also illustrates how GMVs can increase both power and authority by providing a group of patients (and providers) sufficient time to encourage self‐management and by engaging with patients in problem‐solving regarding the day‐to‐day management of a chronic condition:

The health system is not capable of managing so many people and the best way to do it is to have groups with support staff…so you got a team of about five people supporting everybody. If [patients] are intelligent they can figure out how to get the most out of these kinds of groups. They don't have to be pestering their doctor every 10 minutes about some minor thing, [he's] a very busy man.

(Patient Interview #7)

The above mentioned‐excerpts describe a shift occurring where GMVs provided a safe space for patients to increase their own agency, thereby increasing their confidence in managing their chronic conditions.

#### 3.2.2 GMVs help shift power relations between health‐care providers

The analysis of the data also showed that GMVs can shift relationships between providers. Some NPs in the third case were concerned with their role not being visible or valued. Nurse practitioners in the third case described wanting recognition for their work.^64^ Yet, through the process of delivering GMVs in cases 1 and 2, the relationship between the physician and NP shifted. The physician recognized that it was the NP who engaged in the main leadership role. This quote by a physician captures a perspective that runs counter to the notion of doctors having overall authority in the GMV. “The physician is just, just a friendly face in the room…..The nurse practitioner actually takes the main leadership role in our clinic, where she does all the teaching” (Healthcare Provider Interview #12). Additionally, in the GMV where the NP was the only health‐care provider present, the skills and contributions of the NP were recognized by the broader medical community. As this NP stated:

…the outcomes [of the group] became so incredibly successful that the clinics, and then another clinic came on board and just said ‘you know what, however you need to work this, it doesn't really matter what it costs, we're willing to just pay for it’.

(Healthcare Provider Interview #8)

Through the GMV, the understanding of the knowledge and ability of the NP changed amongst the physicians in the community. This same NP described an experience with a diabetic patient who was referred to her. “And so the physician, having no idea what to do with this man next, because he wouldn't do as he was told, sent him to me” (Healthcare provider Interview #8). This interaction represents a shift in the conventional NP/physician relationship, challenging the traditional view of the NP as having less expertise or knowledge than the physician.

## 4 DISCUSSION

This study is unique in its examination of NP‐led GMVs in Canada. Our results suggest an acquisition of knowledge and a disruption of the power differentials between patients and health‐care providers and amongst health‐care providers. Our analysis adds depth to the Diffusion of Innovation Theory^53^ as there has been little consideration of *how* innovations could serve to disrupt existing power differentials.

Patients who attended GMVs described a more engaged sense of communication and increased confidence in managing their condition(s). GMVs also contributed to an environment where the relationships between patients and health‐care providers and amongst health‐care providers become more collaborative and centred around patient needs. Patients attending GMVs had the opportunity to draw on the expertise and care of a NP in addition to harnessing more of their own personal agency to ask questions. Through the GMV, patients also became aware that there were benefits to learning from other patients and listening to health‐care providers interact with other patients.

Past work has shown that GMVs are not necessarily suitable for all patients,^19^ with some indicating that up to 40% of patients approached to attend GMVs declined.^65^ The reasons cited for declining are for legitimate concerns such as being hard of hearing and cognitive deficits. Our work also suggests that gaining more knowledge about a disease trajectory might possibly increase anxiety levels amongst some patients. More work is needed to examine who has attended GMVs, reasons why they may choose to discontinue and whether the GMV has resulted in any unexpected harms to them in terms of gaining more knowledge.

As we described in a previous paper,^64^ NPs in case 3 were working in contexts where they were reluctant to implement GMVs. These non‐adopters of GMVs described aspects of the historical power dynamics that exist between nursing and medicine^37^, ^64^, ^66^, ^67^, ^68^, ^69^, ^70^, ^71^ as barriers to innovation. While NP practice is different from many aspects of registered nursing,^25^ having a nursing background is integral to the professional identity of the NP. Nurse practitioners may encounter many of same challenges associated with power differentials that nurses face,^30^, ^43^, ^66^, ^72^ such as perceptions of NPs as having less knowledge, skills and abilities than physicians. Yet, NPs who were using GMVs described a reconfiguration of these power differentials resulting in NPs having more personal power and authority. In this study, the GMV emerged as a method of care delivery that allowed NPs to harness their professional agency through increased leadership and interdisciplinary collaboration.

This study is not without limitations. We only spoke with patients who had agreed to attend GMVs and were unable to obtain information on the number of patients who had declined to participate. We also only examined two cases of NP‐led GMVs and one case of NPs not using GMVs in BC. More work is needed to examine the use of NP‐led GMVs in other Canadian provinces and jurisdictions. While this study examines the interprofessional processes that can unfold within a GMV, other work has shown that GMVs can positively affect clinical outcomes such as HbA1C and blood pressure, for patients with diabetes.^73^ Although some studies have examined GMVs for heart disease,^74^, ^75^ chronic obstructive pulmonary disease (COPD),^76^ dementia^77^, ^78^ and mental illness,^20^, ^78^ much of the current work has focused on GMVs for diabetes. Finally, we were not able to video or audio‐record the direct observation, so we were unable to complete a more in‐depth analysis of patient‐patient, provider‐patient or provider‐provider interactions. Future work could further examine the impact of NP‐led GMVs for patients who have other chronic conditions, and include patients who did not attend or stopped attending GMVs. Costs associated with GMVs compared to typical consultation visits should also be examined.

Despite the study limitations, this study adds new knowledge on how diffusing new innovations in primary care can disrupt power differentials between patients and providers and amongst providers. Implementing GMVs with the goal of increasing quality of care, particularly for those with chronic conditions, requires attention to power differentials. While there are challenges in diffusing innovations in the complex environment of health care, GMVs create community, encourage interprofessional practice, are patient‐centred and serve to deconstruct some of the traditional hierarchies that exist in primary care.

## CONFLICT OF INTEREST

No conflict of interests has been declared.
