# Supplementary material for: Attending to power differentials: How NP‐led group medical visits can influence the management of chronic conditions
Source: Health Expect. 2017 Jan 10;20(5):862–70. doi: 10.1111/hex.12525 (PMC5600247; doi:10.1111/hex.12525)
Supplement: Supplementary file 1 [file HEX-20-862-s001.docx]

Diffusion of Innovation

**Underpinning theories**

Power

Improved understanding of the complimentary and value added attributes that NPs bring to the care of GMV patients

**Conclusions**

**Draw conclusions from the case studies: Create rich descriptions between and within cases**

**Research questions**

**The case context and phenomenon of interest**

**Refinement of analyzed data through analytic filter**

**Data collection methods**

**Case-study approach**

**Instrumental case-study design**

Direct Observation

**Examining the Impact of NP-led Group Medical Visits for Patients with Chronic Conditions**

**Case-Study Research Questions**

#2) How might GMVs might influence patients’ experiences in primary care?

#3) What is the role of the NP in the delivery of GMVs?

4) What are the barriers and facilitators to implementing NP-led GMVs in BC?

Increased understanding of how GMVs engage patients

Increased understanding of the contexts within which NPs work

Increased understanding of opportunities for NPs to delivery innovative care for patients with chronic conditions

Increased understanding of GMVs impact on patients with chronic conditions

Interviews

**Phenomenon of Interest:** Healthcare delivery for patients with chronic conditions

**Context**: Primary Care practices in British Columbia
